# Incentivizing Commuter Cycling by Financial and Non-Financial Rewards

**DOI:** 10.3390/ijerph17176033

**Published:** 2020-08-19

**Authors:** Vojtěch Máca, Milan Ščasný, Iva Zvěřinová, Michal Jakob, Jan Hrnčíř

**Affiliations:** 1 Environment Centre, Charles University, 162 00 Prague, Czech Republic; milan.scasny@czp.cuni.cz (M.Š.); iva.zverinova@czp.cuni.cz (I.Z.); 2 FEE, Artificial Intelligence Center, Czech Technical University in Prague, 121 35 Prague, Czech Republic; michal.jakob@fel.cvut.cz; 3 Umotional s.r.o., 120 00 Prague, Czech Republic; jan.hrncir@umotional.com

**Keywords:** active mobility, randomized experiment, behavioral change, incentives, smartphone app

## Abstract

Current mobility patterns over-rely on transport modes that do not benefit sustainable and healthy lifestyles. To explore the potential for active mobility, we conducted a randomized experiment aimed at increasing regular commuter cycling in cities. In designing the experiment, we teamed up with developers of the “Cyclers” smartphone app to improve the effectiveness of the app by evaluating financial and non-financial motivational features. Participants in the experiment were recruited among new users of the app, and were randomly assigned to one of four different motivational treatments (smart gamification, two variants of a financial reward, and a combination of smart gamification and a financial reward) or a control group (no specific motivation). Our analysis suggests that people can be effectively motivated to engage in more frequent commuter cycling with incentives via a smartphone app. Offering small financial rewards seems to be more effective than smart gamification. A combination of both motivational treatments—smart gamification and financial rewards—may work the same or slightly better than financial rewards alone. We demonstrate that small financial rewards embedded in smartphone apps such as “Cyclers” can be effective in nudging people to commute by bike more often.

## 1. Introduction

### 1.1. Study Context

Cycling is very popular in the Czech Republic, including in larger cities, where the experiment conducted in this study took place. However, with the exception of several ’flat’ cities in which cycling accounts for up to 20% of commuter journeys in the city (the so-called ’modal share’), bikes are mostly used for sport or recreation. In Prague, for instance, the modal share of cycling in regular commuting is a mere 1–2%; although households possess 2.5 bikes on average, they only possess 1.5 at their Prague place of residence. At the policy level, there is a clearly stated intention to also foster the role of cycling for commuting. The National Cycling Strategy [1] has set the goal of increasing the percentage of travelers using cycling as a mode of transport to 10% by 2020, and the Updated Concept of the Development of Prague Cycling [2] aims to increase the number of cycling residents, equalizing cycling as a regular means of transport and extending the cycling network by 200–500 km by 2020. The development of cycling infrastructure, however, lags behind policy commitments, partly due to other priorities, a complicated regulatory framework, and insufficient funding. Therefore, our research may be of value for providing evidence of motivational measures that may be effective in increasing commuter cycling, long before a well-connected cycling infrastructure will be completed.

This study—a randomized controlled trial—examines whether smart gamification and/or financial incentives are effective in stimulating regular commuter cycling (compared to no incentives), which of these two incentives is more effective, and whether there is an extra benefit of combining these incentives. In addition, this study aims to distinguish the effects per stage of behavioral change, as proposed by contemporary theoretical models, e.g., [3]. Ultimately, our aim is threefold: (1) To foster the app’s effectiveness in supporting behavioral change; (2) to examine the potential effect of different interventions on different segments of users; and (3) to add to the existing knowledge of app-based interventions aimed at increasing physical activity.

### 1.2. Research Rationale

The overarching rationale for this randomized experiment stems from limited (and/or ambiguous) evidence on the effectiveness of incentives for commuter cycling (or physical activity in general) communicated through a smartphone app in changing routine behaviors. In their review, Stewart et al. [4] found little robust evidence of effective interventions for increasing commuter cycling, with the reason for this being that many studies do not use appropriate control groups or have high rates of loss to follow-up. The external validity of these studies has also been limited due to their focus on specific groups of users. Zuckerman et al. [5] reported very similar findings on the effectiveness of gamification for increasing physical activity (such as virtual rewards and social comparisons), and they only found a few rigorously-evaluated studies that yielded contradictory findings.

Interventions using biking apps are also scarce. Wunsch et al. [6] explored three persuasive strategies (a frequent biking challenge, a virtual bike tutorial, and a bike buddy program), Wunsch et al. [7] tested gamification incorporated in a biking campaign, and Bopp et al. [8] tested a multi-strategy intervention using an app alongside a social marketing component and social media campaign. It is worth mentioning that the merits of multi-component interventions are also not entirely warranted. While a review by Baker et al. [9] found no support for the hypothesis that multi-component community-wide interventions effectively increase physical activity, Schoeppe et al.’s [10] review concluded that multi-component interventions appear to be more effective than a stand-alone app, whilst noting that further research is needed.

Furthermore, the evidence on the effectiveness of app-based interventions is rather ambiguous. A systematic review by Baker et al. [9] concluded that while numerous studies on physical activity apps have been undertaken, there is a noticeable inconsistency in the findings, in part confounded by serious methodological issues. Direito et al. [11], in a systematic review and meta-analysis of 21 intervention randomized control trials (RCTs) using mobile technologies to aid public health practices (mHealth), only found a small to moderate, but statistically non-significant, effect on the level of physical activity (PA). Milne-Ives and colleagues [12] systematically reviewed 52 RCT studies and found no strong evidence for the effectiveness of mobile apps because few studies found significant differences between the app and control groups. Han and Lee [13] found that the use of mobile health applications has a positive impact on health-related behaviors and clinical health outcomes.

Zhao et al. [14] reviewed studies on health behavioral change using mobile phone apps and found that of 23 studies in total (all conducted in high-income countries), 17 studies reported statistically significant effects in the direction of targeted behavioral change, while six studies reported using behavioral change theories. Self-monitoring was the most common behavioral change technique applied (in 12 studies). Payne et al. [15] reviewed 24 studies—primarily feasibility and pilot studies—and called for large sample studies using mobile phone apps for a more rigorous evaluation of efficacy and establishing evidence for best practices.

A review by Yang et al. [16] observes that contemporary physical activity apps have implemented a limited number of behavioral change techniques (BCTs), with the most frequent being social support, information about others’ approval, instructions on how to perform a behavior, feedback on behavior, goal setting, and prompts/cues. In their review of 25 studies, McDermott et al. [17] aimed to identify BCTs associated with changes in intention and behavior, and reported medium-to-large effects on intentions and small-to-medium effects on behavior, but failed to produce evidence on how to facilitate behavioral change through a change in intention. 

In contrast, personal financial incentives have been shown to be effective in increasing the attainment of target levels of health-related behavioral change (although weakening over time, cf., [18]). Still, the incentives reviewed by Mantzari and colleagues [18] differed widely in their nature, with direct monetary payments or quasi-monetary lottery tickets, such as gift certificates or vouchers; in the modality of rewards, i.e., lump-sum payments, payments or deposits released per unit of achievement; and in the certainty of rewards, i.e., lottery vs. certainty. Furthermore, there are not many rigorous evaluations of existing fiscal incentives for commuter cycling, such as the study by de Kruijf et al. [19] on e-cycling. This is somewhat surprising given that various incentives are provided on a broad scale to employees, including cycling allowances in Belgium, the tax-free provision of bikes in the Netherlands and the UK, and direct rewards for cycling to work recently introduced in Bari, Italy.

## 2. Materials and Methods

### 2.1. Study Design

**Figure 1:**
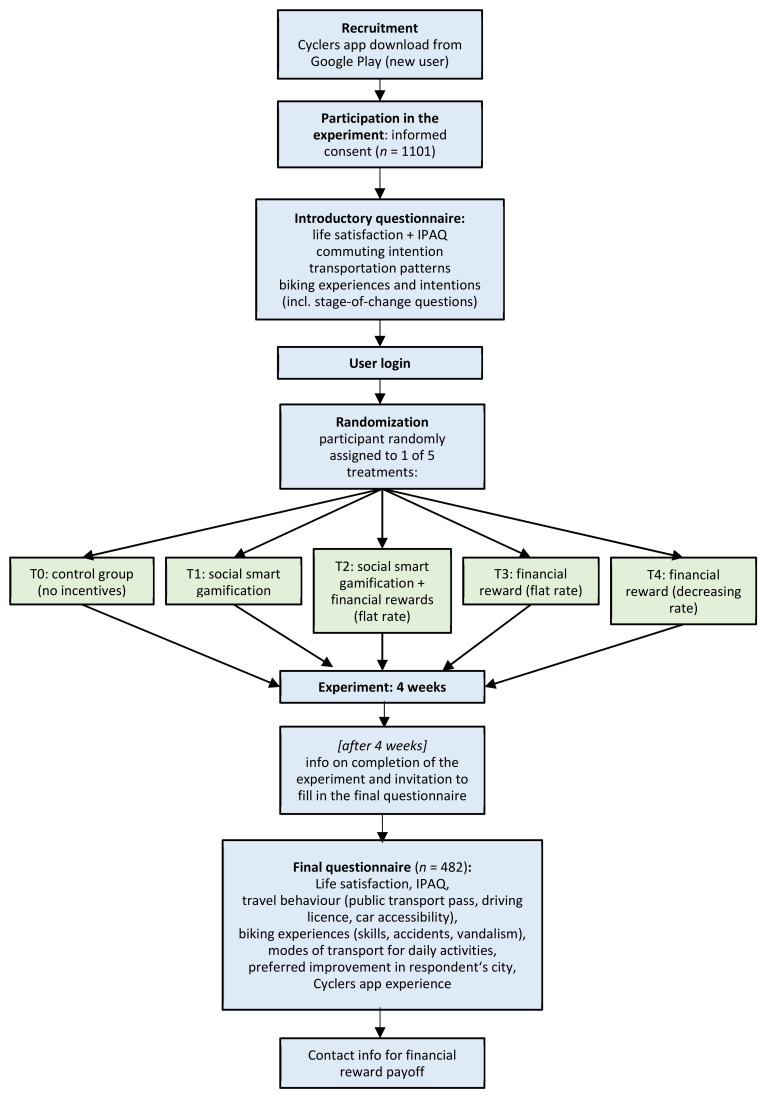
Flow diagram of the study.

Randomized controlled trials are considered the most rigorous way of determining whether a cause–effect relationship exists between a treatment and outcome [20]. By randomizing subjects into groups, we eliminated potential selection bias and allowed for statistical analyses to be conducted for comparable independent groups [21]. Our randomized experiment features two motivation incentives: Smart social gamification and financial rewards. However, the design is more complex, with five arms in total (labeled as T0 to T4 in Figure 1), in order to elucidate the extra benefits of a combination of incentives and examine rewards varying in terms of the financing profile (cf. flow diagram in Figure 1). Each participant is attributed to one of the five groups at random.

The smart social gamification of participants in treatment arms T1 and T2 consists of the app’s built-in system of points, badges, leader-boards, and challenges, combined with personalized push and in-app notifications.

Financial rewards are offered to participants assigned to any of the T2-T4 treatment arms. There were two distinctive profiles of reward rates: A flat-rate and a decreasing block rate. In the flat rate profile, participants were rewarded CZK 1 for every kilometre cycled to work/school (and capped at CZK 500, i.e., approx. €20). In the decreasing block rate, each subsequent 100 kilometres traveled was rewarded at a lower rate (ranging from CZK 3/km for 1 to 100 km to CZK 0.2/km for 401 to 500 km, and the maximum reward was capped at CZK 670, approx. €26). These rewards were paid in cash (effectively sent to participants’ accounts) after completing the final questionnaire, including providing necessary account details.

It has been repeatedly emphasized in the literature that interventions should be based on a more thorough understanding of the psychological processes underlying a behavioral change, i.e., viewing it as a transition through a sequence of different discrete stages [3,22,23]. This has practical consequences in that, instead of one single intervention designed for all people, specific intervention packages should be matched to the needs and barriers of people in specific stages. Examples of such models are the stage model of self-regulated behavioral change [22,24] or various modifications of the Transtheoretical Model of Behavioural Change (TTM), such as the model of action phases [25].

Practical examples of these developments include Bopp et al. [8] combining TTM and social cognitive theory targeting behavioral constructs of self-efficacy, self-regulation, outcome expectations, and processes of change. Thigpen et al. [3], using the Model of Action Phases, found that travel attitudes matter more to progression toward regular commuter cycling than travel attributes, thus tentatively supporting the efficacy of soft policies focused on changing travel attitudes.

In our study, a set of questions adapted from Thigpen et al. [3] was used to distinguish the individual stage-of-change of each participant (Table 1).

Subsequently, the in-app notifications were adapted to broadly reflect the stage of change of participants. The following types of prompts were sent (by treatment arms):

- To those who registered for the experiment, but did not record any ride within two (three) days after the registration (T1 + T2):
  ○ Infrequent bikers—messages promoting the benefits of regular biking, and
  ○ frequent bikers—messages promoting the gamification features of Cyclers;
- First (third) ride recorded (T1 + T2)—congratulations for recording the first ride;
- First badge (T1 + T2)—congratulations for the first badge (after 10 rides);
- Weekly summary information (T2–T4):
  ○ If at least one ride recorded—message detailing the amount of financial reward secured so far and the number of days to the end of the experiment, and
  ○ if no rides recorded—message reminding the user about the financial reward awarded per kilometre and the number of days to the end of the experiment.

No notifications were sent to participants in the control group, and only weekly summary information was sent to participants in T3 and T4 treatments. After 4 weeks, all participants (including those in the control group) were invited to complete the final on-line questionnaire by an in-app notification and e-mail.

This particular setup of the experiment was tailored to allow us to discern what incentives are effective in which stage-of-change, i.e., to suggest when, how, and to whom such incentives can be effectively targeted and what effects may be expected.

In the final questionnaire, we asked questions already included in the short introductory questionnaire on the participant’s life satisfaction and level of physical activity (using a short form of the International Physical Activity Questionnaire (IPAQ)). We asked questions about regular travel behavior (transport modes used for specific purposes, such as commuting to work or school, for shopping, and for leisure activities), possession of a public transport pass, possession of a driver’s license, and car availability. We also asked respondents about their cycling experiences (skills, accidents, and vandalism), perceived barriers to cycling, and what kind of improvement(s) for cyclists they want implemented in their city the most. In addition, we asked about their mode of use of the Cyclers app and the user experience of the app.

### 2.2. Recruitment of Participants and Data Collection

Cyclers is a cycling smartphone application developed to promote regular biking in cities. It focuses on facilitating and motivating self-regulated behavioral change by providing various planning tools, feedback, rewards, and experience sharing. Its key features include a cycling route planner (as of now with full coverage of Europe and North and South America); turn-by-turn navigation that allows for combining biking with public transport; and route tracking that is linked to a system of badges, challenges, and rewards and community experience sharing. The routing engine is based on state-of-the-art artificial intelligence algorithms that allow users to set preferences for various route optimization criteria, including safety, comfort, and physical exercise. In Czechia, the app is also linked to the country-wide Bike-to-Work campaign that targets employees and offers several competition categories, including the number and total length of bike trips. In short, Cyclers is an app that focuses on facilitating and motivating self-regulated behavioral change. To that end, the users’ exposure to active mobility/physical activity and nature-contact is increased and habitualized.

The Cyclers app was adapted to the RCT design described above, both in the app’s frontend (screen features) and backend (database). Once the programming was completed, a thorough pre-testing of the modified app and data transfers from the app to the final questionnaire were conducted. Upon her/his agreement to participate, each participant was given a unique ID that subsequently featured in a link to the final questionnaire that the participant was asked to complete. Once the participant opened the final questionnaire, a call was sent to the Application Programming Interface (API) of the Cyclers app using the unique ID. The response to this API call was a set of data from the app database consisting of the user’s nickname, email, treatment group, number of rides, kilometres traveled, and financial reward accumulated (except for T0 and T1, where no financial rewards were offered).

Participants in the experiment were recruited from among those who downloaded the Czech version of the Cyclers app from the Google Play store (new users), and upon their consent to participate in the experiment, they were randomly assigned to one of the treatment arms (i.e., either to one of the treatment groups or the control group). Initially, no specific promotion of the experiment was planned, but due to the very low conversion rate observed (i.e., enrolment in the experiment), an invitation to download the app and participate in a scientific project was posted to several websites and Facebook groups. All instructions related to participation in the experiment (along with informed consent) were contained in the app. This research is organized by Charles University Environment Centre in cooperation with Umotional Ltd. (developer of the UrbanCyclers application) as one of the activities of the INHERIT project (International Health and Environment Research for InnovaTion, grant no. 667364) funded by the European Union. The project of this research was approved by the Institutional Review Board of Charles University Environment Centre.

We estimated the optimal sample size for an experiment with five treatment arms with a conservative assumption of a small effect (*d* < 0.15), but conditional on how many participants we effectively managed to recruit in the given time frame of the study. Given the low conversion rate (i.e., the ratio between those who downloaded the app and who subsequently enrolled in the experiment) that we encountered in the summer/autumn of 2018 and the remaining time frame for data collection during spring 2019, we aimed to obtain a minimum sample size (still sufficient for disentangling an effect of size *d* = 0.16) of about one hundred participants per treatment arm, i.e., 500 participants in total.

### 2.3. Statistical Methods

Our primary goal was to determine whether any of the motivational features induce more commuter cycling. To do so, we took the number of rides to work or school recorded by each participant in the app during the experiment as the explained variable (i.e., outcome) and the treatment variant as the explanatory variable. As some of the participants may have no commute rides recorded, while others will have more than 20 rides, a model for count data allowing for over-dispersion should be used in such an analysis. Therefore, we opted for the negative binomial regression model as our initial model.

The basic model equation may be described as

(1) $$rides=exp\left(intercept+b_{1}\left(treatment=sGam\right)+b_{2}\left(treatment=sGam+fRate\right)+b_{3}\left(treatment=fRate\right)+b_{4}\left(treatment=dRate\right)\right),$$

where the outcome (i.e., the number of rides recorded by a participant) is predicted with a linear combination of variants of treatment: *sGam* is T1 (smart gamification), *sGam + flRate* is T2 (smart gamification with a flat rate financial reward), *fRate* is T3 (flat rate financial reward), and *dRate* is T4 (decreasing block rate financial reward). Taking the control group (T0) as a reference, we estimated one coefficient for each treatment (*b*’s in the formula). The effects of treatments in the model are additive to the reference, so if any of the coefficients are statistically significant, it captures the effect of this particular motivational feature (or their combination).

To take into account that a certain number of participants did not record any ride in the experiment, we further used a regression structure that considered participants with zero rides through a different generation process compared to positive counts (rides). In our case, the zero-inflated negative binomial regression model simultaneously ran two equations: A binary equation to model the zeros in the outcome variable and a count data estimation to model the positive count of rides.

## 3. Results

### 3.1. Study Characteristics

Table 2 summarizes the descriptive characteristics of the participants in the experiment. Overall, there is a marked overrepresentation of male participants (63%), with a high education (38%) and employed (69%), but this clearly reflects that urban cycling is more frequent among males and that we targeted people who commute by bike to work or school. On average, our participants were about 38 years old and lived in a household comprising three members, with one being a child under the age of 18.

**Figure 2:**
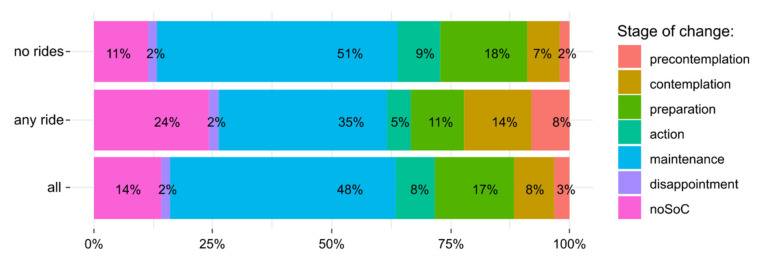
Classification of participants according to the stage-of-change. “noSoC” denotes participants who provided insufficient information for stage-of-change allocation.

Figure 2 depicts the allocation of participants to respective stage-of-change classes. Since one fifth of our sample (*n* = 99) did not record any ride, we report stage allocation separately for those who did not record any ride (“no rides”), those who recorded at least one ride (“any ride”), and all participants together (“all”).

### 3.2. Effectiveness of Incentives 

**Figure 3:**
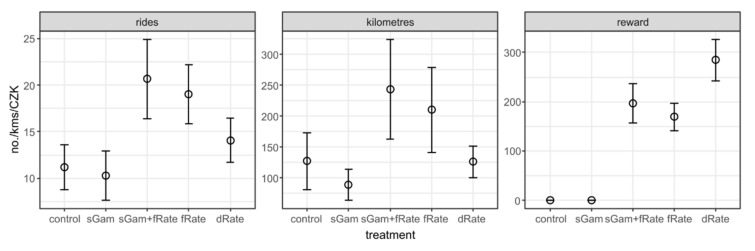
Summary statistics on the number of rides, total length of rides in kilometres, and total financial rewards in CZK per participant. Note: sGam is T1 (smart gamification), sGam + flRate is T2 (smart gamification with a flat rate financial reward), fRate is T3 (flat rate financial reward), and dRate is T4 (decreasing block rate financial reward).

Figure 3 provides summary statistics on the number of rides to work or school recorded during the experiment, total kilometres cycled, and rewards earned per treatment variant. In T1 (smart gamification treatment), the mean and median number of rides recorded was 11.2 and 3.5; in T2 (smart gamification with a financial reward), 20.7 and 16; in T3 (flat rate financial reward), 19 and 17.5; and in T4 (decreasing block rate reward), 14.1 and 11. In the control group (T0), the mean number of rides was 11.2 (and the median was 5). The total sum of kilometres cycled to and from work or school during the experiment was the highest in T2 (mean 244 km, median 168 km) and T3 (mean 210 km, median 134 km), and the lowest in the control group (mean 127 km, median 25) and T1 (mean 88.7 km, median 31 km). The reward earned per participant (relevant in T2, T3, and T4) was the highest in T4 (mean CZK 285, median CZK 279), followed by T2 (mean CZK 197, median CZK 165), and comparatively the lowest in T3 (mean CZK 169, median CZK 134).

**Figure 4:**
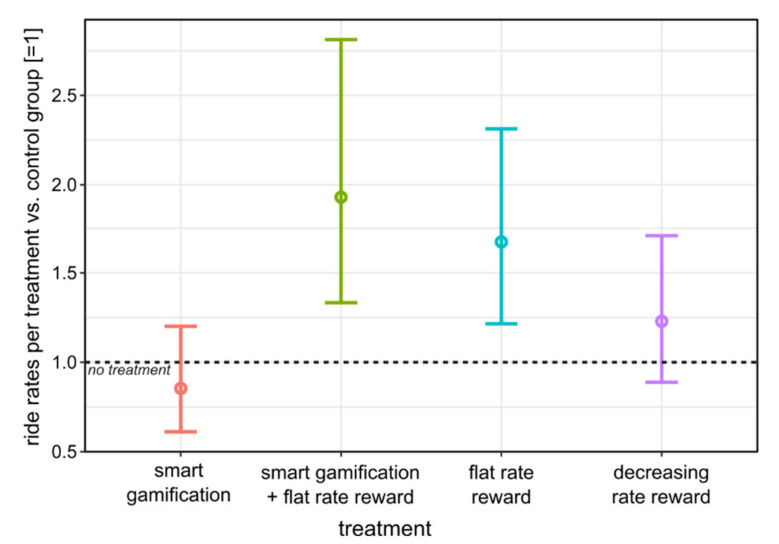
Predicted probabilities of the frequency of rides (vs. control group T0).

To further analyse the effect of treatments, we estimated the negative binomial regression model described earlier. Figure 4 shows the estimated coefficients, along with their 95% confidence intervals, in order to document the effect of treatment vis-à-vis the control group (dashed line at 1). The most effective incentivization is obtained in the treatment with combined smart gamification and flat rate rewards (T2), which has almost doubled the number of commuter cycle rides. The provision of flat rate rewards (T3) is predicted to increase the number of rides by two thirds. The provision of decreasing block rate rewards (T4) leads to a small increase in the number of rides, but the effect is not statistically significant (for a commonly used 5% level of significance). Finally, smart gamification treatment (T1) is predicted to slightly reduce the number of rides compared to no treatment, but again, this effect is not statistically significant.

Due to the substantial number of participants with zero rides, we further estimated a zero-inflated negative binomial regression model (cf. Table 3), in which we also controlled for participation in the Bike-to-Work campaign (Bike2Work).

This model fits the data better than a simple negative binomial model (χ^2^ test *p* < 0.001), reflecting a substantial proportion of participants with zero recorded rides. In addition, the logarithm of θ (an inverse of alpha) is significant, confirming the overdispersion. The inflated coefficients that are significant suggest that those respondents who were participating in Bike-to-Work were much more likely to record a nonzero number of rides to school or work in the Cyclers app during the experiment (which is a rather trivial but assuring observation), while those who were attributed to preparation, action, and maintenance stages of change were more likely to record some rides, as well as those who were allocated to any of the three experimental treatments with financial rewards (flat rate, flat rate with smart gamification, and decreasing block rate).

In the negative binomial regression part, the model suggests that both experimental treatments with flat rate rewards (i.e., flat rate and flat rate with smart gamification) statistically significantly increase the number of recorded rides. The interaction terms of stage-of-change with engagement in Bike-to-Work are significant and positive for four classes of stage-of-change: Contemplation, preparation, maintenance, and unidentified. This is in line with our expectation that the participants in Bike-to-Work are either occasional or regular commuter cyclists. We would expect the same to be true for participants attributed to the action stage, where the coefficient is also positive, but not significant, perhaps due to the small number of those attributed to this stage.

### 3.3. Enablers of and Barriers to Commuter Cycling

**Figure 5:**
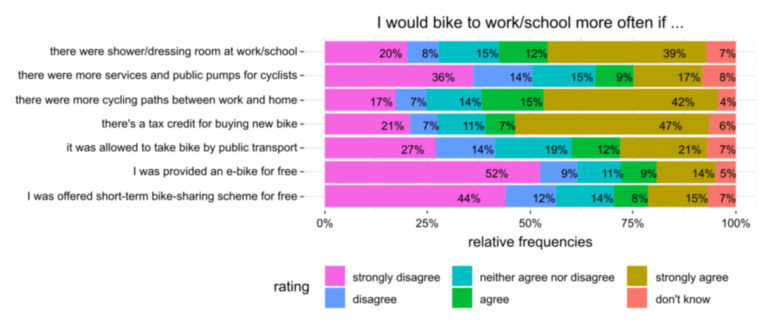
Perceived enablers of more frequent commuter cycling.

In the final survey, we explored what “enablers” may alleviate the pursuit of more frequent commuter cycling. Our respondents (as shown in Figure 5) indicated that the provision of facilities such as showers and dressing rooms at work, better cycling infrastructure, and financial incentives for the purchase of new bikes would increase their likelihood to engage in frequent commuter cycling.

Among the various barriers, the respondents were particularly concerned with exposure to bad weather (64% deemed it likely or very likely); the need to accomplish more tasks during the day (36% deemed it likely or very likely); a need to carry along more belongings (27% deemed it likely or very likely); and to a somewhat lesser degree, the risk of being involved in a traffic accident (15% deemed it likely or very likely).

## 4. Discussion

This study fits into a growing stream of mHealth research aimed at influencing physical activity and sedentary behavior. Daily commuting to work (or school) is a prominent candidate for such an intervention, which may not only improve one’s health, but also has a potential to improve the liveability of cities by reducing car use and ownership.

Using a randomized experimental design, we compared the effects of the provision of monetary and non-monetary incentives on the frequency of commuter cycling. The strength of our study stems from its rigorous approach of a randomized experimental design and use of convenient and, to a large extent, unobtrusive, data collection through a smartphone app, without any need for a face-to-face encounter between researchers and study participants.

In this respect, we demonstrate that a smartphone app can be used as a means of intervention, despite the fact that we observed similar difficulties to previous studies, such as a rather low rate of participants’ enrollment and substantial drop out of participants from the study, e.g., [8,26,27].

The key finding of this study is that small monetary rewards for each kilometre cycled to work (or school) can effectively motivate one to significantly increase the frequency of cycling. While this finding may be warmly welcomed in Bari and other cities pondering the incentivization of cycling by monetary rewards, the limited scope and duration of the experiment prevent the inference of any long-term effect or persistence of an effect after incentive cessation. Nevertheless, a similar study aimed at switching car commuting to e-bikes in the Netherlands using monetary incentives found not only a significant positive effect after one month, but also a further increase in cycling after six months [19]. Our results also provide a useful insight into a rewards structure in that a reward with a relatively modest flat rate outperforms a decreasing block rate that starts several times higher.

We found a modest increase in the ride frequency among participants in the treatment group with both financial and nonfinancial incentives compared to the treatment group with only a financial incentive. Even though the combination of financial and non-financial incentives may not be considered a real multi-component intervention, it is a finding that is rather supportive of the observation in Schoeppe et al. that multicomponent interventions appear more effective [10]. This is also evident from the unintentional concurrence with the Bike-to-Work campaign in May 2019 that increased the number of rides even more than our experimental incentives (but also in a short time-span). All of this, in conjunction with the importance of various enablers of and barriers to more frequent cycling reported by our respondents, clearly shows that the effective promotion of commuter cycling is a multifaceted endeavor that requires an integrated package of many different complementary interventions [28].

In contrast to financial incentives, we found no effect of smart gamification on the frequency of commuter cycling. This seems to corroborate findings from a similar gamification study [5] that warns against the simple assumption that gamification is always an effective approach for promoting opportunistic physical activity. As gamification often encompasses different elements (points, badges, leader-boards, and challenges in our case), it may be worthwhile to evaluate each of these elements separately.

Although the arbitrariness of stage-of-change boundaries has been widely discussed [29], the allocation of participants to slightly adapted stages proves useful in explaining active participation in the experiment (i.e., non-zero number of rides) and also in disentangling the contributing effect of the Bike-to-Work campaign, only pronounced in occasional and regular cyclers and not among those in pre-contemplation and disappointment stages. This clearly points to a need for interventions and incentives tailored for respective groups that will enable them to move further along the stage-of-change path.

Nevertheless, the limitations of the study are clear. First and foremost, it suffers from a limited scope and short duration, and therefore, the observed effect on commuting behavior is only a short-term effect. Acknowledging the limited sample size and its non-representativeness, the findings are rather tentative, and with a larger sample, it would definitely benefit from more elaborate analyses, e.g., controlling for various sociodemographic, socio-economic, and other contextual factors that have been shown to influence commuting behavior, such as gender, age, family and occupational status, bike availability, biking skills, and perceived safety [30,31,32]. Both of these limitations point to a challenge for future research: These studies should examine potential long-term effects, the persistence of an effect after incentive cessation, and additional means to habitualize induced behavioral change.

## 5. Conclusions

Smartphone-based interventions in the public health domain are still a rather novel approach and their potential has not been fully developed. While we have demonstrated that these interventions can be used with relatively limited resources for a short period of time, it is crucial to explore long-term consequences of such efforts. Many daily tasks, commuting included, are habitualized and short-term interventions may not break these routines. In addition, it would be beneficial to compare alternative modes of intervention delivery (e.g., via social networks or regular phone calls).

Thanks to the ubiquity of smartphones and people’s attachment to them, particularly among the younger population, it is rather easy to transfer and scale-up such an app-based intervention to different cities and/or countries. There is, however, a ’mode-of-delivery’ question to consider, i.e., how this can be done most effectively. One way is to use dedicated niche apps (such as Cyclers) and to motivate the broader public to install and use them; another is to embed motivational features into apps already used by a large part of the population (such as Google Maps or Facebook).

In terms of possible transferability hurdles, bicycle availability and the ability to cycle might be obstacles, perhaps more so among people with a lower socioeconomic status. One option here is to build upon the growing availability of shared bikes, and to combine rewards with some form of bike-sharing programme subscription (or include a bike-sharing option in a public transport pass).

Ultimately, a crucial question for policymakers to resolve relates to what the role of smart ’pull’ incentives (such as financial incentives for commuter cycling) should be in the entire policy mix aimed at redesigning our urban transport systems into healthy, carbon-free, and affordable ones. Placing the promotion of cycling at the top of this agenda has been demonstrated to pay off not only in terms of improved public health, urban environments, and sustainable mobility, but also in terms of green jobs [33].

## Figures and Tables

**Table 1:** Stages-of-change classification.

Survey Question	Stage Allocation					
Did you go to work/school last week at least once on a bike?	Did not bike in past week	Did not bike in past week	Did not bike in past week	Biked at least once in past week	Biked at least once in past week	Biked at least once in past week
What mode of transport do you usually use to travel to work/school?	Other	Other	Other	Other	Bike	<any>
Have you thought about biking to work/school?	No	Yes	Yes	Not asked	Not asked	Not asked
How likely are you to go to work/school at least once by bike in the next 4 weeks?	Not likely	Somewhat likely	Very likely	(very) likely	(very) likely	Not likely
Stage of change	Pre-contemplation	Contemplation	Preparation	Action	Maintenance	Disappointment

**Table 2:** Descriptive statistics of study participants (*n* = 482).

Indicator	Mean (SD) or Pct.
Age	37.7 (9.4)
Gender	
- Female	37%
- Male	63%
Education level	
- Low	26.8%
- Middle	35.3%
- High	37.8%
Household size	3.1 (1.2)
Children in household	0.9 (1.0)
Economic activity	
- Employed	69%
- Self-employed	4%
- Student	5.5%
- Other/not disclosed	21.5%
Participation in Bike-to-Work	35.7%

**Table 3:** Zero-inflated negative binomial regression model for rides to work/school recorded in the experiment (dependent variable: number of rides to work or school recorded in the app during the experiment).

Variable	Estimate	Std. Error	*z*-Value
Count Model (Rides > 0)			
Constant	2.305 ***	0.389	5.933
SoC: contemplation	−0.509	0.432	−1.178
SoC: preparation	−0.453	0.411	−1.104
SoC: action	0.201	0.424	0.475
SoC: maintenance	0.385	0.388	0.993
SoC: disappointment	0.268	0.526	0.510
SoC: missing	−0.116	0.413	−0.282
treatment: sGam	−0.048	0.145	−0.333
treatment: sGam + fRate	0.339 *	0.141	2.403
treatment: fRate	0.253 *	0.124	2.033
treatment: dRate	0.049	0.130	0.379
SoC: precontemplation × Bike2Work	0.295	0.612	0.482
SoC: contemplation × Bike2Work	1.416 ***	0.344	4.114
SoC: preparation × Bike2Work	1.149 ***	0.207	5.547
SoC: action × Bike2Work	0.328	0.287	1.145
SoC: maintenance × Bike2Work	0.414 ***	0.118	3.507
SoC: disappointment × Bike2Work	0.431	0.680	0.635
SoC: unidentified × Bike2Work	0.882 ***	0.257	3.426
Log(θ)	0.512 ***	0.089	5.746
**Zero-inflation model (rides = 0)**			
Constant	1.215.	0.693	1.754
SoC: contemplation	−1.086	0.795	−1.366
SoC: preparation	−1.653 *	0.807	−2.049
SoC: action	−2.303 *	0.904	−2.546
SoC: maintenance	−1.918 **	0.709	−2.707
SoC: disappointment	−1.523	1.140	−1.337
SoC: unidentified	−0.694	0.746	−0.93
treatment: sGam	0.360	0.367	0.979
treatment: sGam + fRate	−1.770 **	0.618	−2.863
treatment: fRate	−1.382 **	0.457	−3.021
treatment: dRate	−1.674 ***	0.481	−3.481
Bike2Work	−3.473 ***	0.811	−4.282

Notes: SoC—Stage-of-Change. Signif. codes: ***, <0.001; **, <0.01; *, <0.05; and “.”, <0.1.
